# Attrition in a Multi-Component Smoking Cessation Study for Females

**DOI:** 10.1186/1617-9625-3-2-59

**Published:** 2006-08-15

**Authors:** Robert F Leeman, Zandra N Quiles, Laurence A Molinelli, Donna Medaglia Terwal, Beth L Nordstrom, Arthur J Garvey, Taru Kinnunen

**Affiliations:** 1 Department of Psychiatry, Yale University School of Medicine, New Haven, CT; 2 Lehman College, City University of New York, New York, NY; 3 Tobacco Dependence Treatment and Research, Harvard School of Dental Medicine, Boston, MA; 4 Ingenix Pharmaceutical Services, Newton, MA

## Abstract

Limiting attrition (i.e., participant dropout before the conclusion of a study) is a major challenge faced by researchers when implementing clinical trials. Data from a smoking cessation trial for females (*N *= 246) were analyzed in order to identify baseline smoking-related, demographic and psychological characteristics affecting likelihood of early (i.e., before the quit attempt) and late (i.e., after the quit attempt) dropout. There were a number of significant demographic predictors of attrition. Participants with at least one child living at home were at increased risk of both early and late dropout. Non-Whites were at increased risk of early dropout, while not having a college degree put one at increased risk of late dropout. Age was found to be a protective factor in that the older a participant was, the less likely she was to drop out in the early stages of the trial. With respect to psychological variables, weight concerns increased risk of attrition, as did the experience of guilt. In terms of smoking-related variables, mean cigarettes per day was not a significant predictor of attrition, although length of longest prior quit attempt was a significant predictor of early dropout when age was removed from the regression.

## Introduction

Participant attrition (i.e., participant dropout before the conclusion of a study) is a threat to the validity of research findings in that attrition introduces sampling bias. Attrition also hurts the cost-effectiveness of research because limited staff time and financial resources are likely to be invested in "dropouts," who typically yield little to no useable data [1]. Recognizing the importance of attrition, researchers have begun to suggest that analyses should be conducted with completion of treatment as an outcome variable in addition to abstinence [2]. Given the many contrasting and equivocal findings in the small literature on this topic, limited progress has been made up to this point in identifying the characteristics associated with attrition.

In the attempt to explain rising attrition rates and the relatively low percentage of successful quit attempts in smoking cessation trials, researchers have put forth the notion that remaining smokers in the population are primarily heavily addicted, long-term users [3,4], who are resistant to the treatments presently available [5,6]. Females likely comprise a high percentage of this heavily addicted, long-term population of smokers. While there is debate in the literature as to whether or not women have had greater difficulty with smoking cessation during the past several years [7-13], there is clear evidence that gender differences in smoking prevalence declined during the second half of the 20^th ^century with more women than men taking up smoking [14]. Accordingly, a precipitous drop in sex mortality ratios for lung cancer could also be observed during the latter half of the 20^th ^century, a decline from a 6.7 to 1 male/female ratio in 1960 to 2.3 to 1 in 1990 [14].

Several smoking-related variables have been analyzed for their effects on attrition. Among a sample of female smokers [15] and smokers with a history of depression [3], a high number of cigarettes smoked per day (CPD) has been found to be associated with missed study meetings and attrition. However, in a small study involving Hispanic smokers (*N *= 93), Nevid et al. [16] found null results with respect to CPD. Length of prior quit attempts and confidence that one will succeed have also been found to influence attrition. Borrelli et al. [2] found that female smokers with prior quit attempts of a longer duration were less likely to drop out and both Munoz, Marin, Posner and Perez-Stable [17] and Nevid et al. [16] concluded, perhaps counter intuitively, that Hispanic smokers with higher confidence in the success of their quit attempt at baseline were more likely to drop out.

In terms of demographic predictors, there is indirect evidence suggesting that participant race may influence attrition. Nevid et al. [16] reported a tendency on the part of minorities to terminate outpatient community-based treatment services sooner than Whites. In addition, there is an oft-reported finding that members of minority groups tend to be distrusting of medical research, perhaps due to the mistreatment minorities have received in past medical research [18]. Other demographic variables that may increase likelihood of attrition include lower education level [2,3], (although Nevid et al. [16] found null results) and a lower Body Mass Index (BMI) [2,19]. Given that female smokers are likely to have competing family responsibilities that may interfere with their commitment to cessation treatment [20], having children currently living at home may increase likelihood of attrition. There are also findings that point to a relationship between age and attrition. Fortmann and Killen [8] found that enrolled participants in their study tended to be older than qualified non-participants, suggesting that older individuals may be more committed to cessation and accordingly, less likely to drop out of studies. However, Curtin et al. [3] and Nevid et al. [16] reported no significant relationships between attrition and age.

A psychological variable that would seem likely to influence attrition is concern about weight gain since women frequently report weight control as a reason for smoking [21,22]. Borrelli et al. [2] found no significant relationships between weight concerns and attrition though. In light of the abstinence violation effect [23] – the occurrence of a "slip" during a cessation attempt, which the participant attributes to internal, stable and global factors (e.g., personal weakness) – a prediction could be made that tendencies toward guilt would be relevant to attrition. According to this scenario, a temporary slip during a quit attempt would be accompanied by intense feelings of guilt. Those who are more prone to guilt may experience particularly strong feelings should such a slip occur. Due to these feelings, "high guilt" participants may decide to drop out rather than magnify their guilt by admitting to researchers that they have smoked.

Data from a clinical trial evaluating exercise as an adjunctive treatment for nicotine gum among female smokers were analyzed in order to determine which participant characteristics were associated with attrition. An analysis such as this may help to clarify which smokers may be at heightened risk for attrition and potentially which attributes should be addressed by researchers in order to keep more participants in trials and make their samples more representative of the smoking population.

## Method

### Participants

Participants were recruited from the greater Boston, MA area using a combination of radio, newspaper and television advertising. In these advertisements, female smokers were invited to participate in a quit smoking study in which they would receive free nicotine gum and additional treatment should they qualify.

The main exclusion criteria were current involvement in exercise at least once per week, an average consumption rate of fewer than five cigarettes per day, active and severe psychiatric illness, a history of a serious vascular or cardiac condition, bleeding peptic ulcers and insulin-dependent diabetes mellitus. Participants with conditions contraindicated with nicotine gum use such as temporomandibular joint disorder, bleeding peptic ulcers, pregnancy or lactation were excluded as well. Information regarding exclusion criteria was obtained from a preliminary telephone screen and a baseline clinical diagnostic exercise test/medical screen.

### Procedures

This clinical trial was approved by the Office for Research Subject Protection at Harvard Medical School and the Human Research Committee at Brigham and Women's Hospital. All participants were provided with nicotine gum treatment and brief behavioral counseling and were randomized into one of three conditions each lasting a total of 19 weeks (three weeks pre-quit and 16 weeks post-quit). The first was an exercise condition consisting of 40 minute supervised sessions including a five-minute warm-up routine, 30 minutes of aerobic exercise (i.e., walking or running on a treadmill), followed by a five-minute cool down. Participants in this condition were also strongly encouraged to take part in home-based exercise sessions and several suggestions (e.g., walking) were offered. The second was an equal-contact control condition consisting of health and wellness lectures. These sessions were of equal duration as the supervised exercise sessions. These wellness sessions included no tangible cessation help beyond the brief behavioral counseling that all participants received. The third was a standard care control condition involving no additional treatment beyond the nicotine gum and brief behavioral counseling. No significant differences based on condition assignment were found for any variables assessed at baseline.

Those randomized into the exercise and equal contact control wellness conditions were asked to attend twice weekly sessions for five weeks followed by weekly sessions for the remaining 14 weeks. Participants in the standard care condition were provided eight sessions over the course of the 19-week study. All but one of these sessions took place following the quit date. All sessions were held in the late afternoon/early evening and session locations were in close proximity to public transportation. Participants were given a payment of $50 upon completion of the study.

### Measures and Analyses

A number of smoking-related items were included in the baseline questionnaire, including mean cigarettes smoked per day (CPD), duration of longest prior quit attempt, number of years as a smoker and nicotine dependence, which was measured using the revised Fagerstrom Test for Nicotine Dependence (FTND) [24].

The following demographic characteristics were analyzed: race, highest level of education, body mass index (BMI), age and number of children currently living at home.

In terms of psychological variables, confidence in the quit attempt was assessed with a single item rated on a five-point scale from "very slightly or not at all" to "extremely." Concern about postcessation weight gain was assessed using a scale developed by Borrelli and Mermelstein [25], made up of six items measured on five-point Likert scales (e.g., "How likely is it that you would go back to smoking after quitting if you gained too much weight?") (an alpha of .87 was provided by the authors). Depression was assessed with the Center for Epidemiological Studies Depression Scale [26]. The guilt scale was comprised of five items from the Personal Feelings Questionnaire-2 Revised Scale [27] assessing a series of feelings and behaviors (e.g., "I felt intensely guilty") for which participants were to rate their frequency of occurrence during the past week on a four-point scale (alpha = .71 from the present study).

Participants who met the following three qualifications were included in analyses: 1. passed both the preliminary telephone screen and the baseline clinical diagnostic exercise test/medical screen; 2. were able to be contacted regarding their treatment group randomization and 3. provided complete baseline data. Similar to the approach used by Curtin et al. [3], participants were divided among three participation levels: 1) early dropouts, who dropped out before making a quit attempt; 2) late dropouts, who made a quit attempt but did not complete treatment and 3) treatment completers. Minimal criteria for treatment completion were attendance at one of the final two assessments (week 12 or 16) and absence from no more than two of the nine assessments conducted during the 19 weeks of the trial.

Correlation coefficients were used to assess relationships among all predictor variables and to identify highly correlated variables (i.e., over 0.50) for the purposes of preventing multicolinearity in the main analysis. All variables were entered into a preliminary multinomial logistic regression. In cases of highly correlated pairs, the weaker predictor was excluded from the main analysis. *T*-tests were planned to assess whether participants who differ with respect to the dichotomous demographic classifications also differ with respect to any continuous predictor variables that significantly predicted attrition (e.g., racial differences in guilt scores).

Multinomial logistic regression was the main method of statistical analysis, using participation level as the outcome measure with treatment completer as the reference group. Treatment group assignment was also included as a predictor variable because of the possibility that assignment to either the standard care control or equal contact control conditions would be associated with a greater likelihood of attrition. The lack of definitive findings in the attrition literature did not justify hierarchical entry of variables into the regression, thus all predictor variables were entered simultaneously.

Race was dichotomized into White/Non-white, education into graduated/did not graduate from college and number of children at home into have none/have at least one. A log transformation was conducted in order to correct a large positive skew in the distribution of the length of longest prior quit attempt variable. For all continuous variables, a value three standard deviations above the mean was calculated and scores higher than this value were rounded down to this cutoff point to prevent undue influence on the part of outliers.

## Results

### Description of Sample

Six prospective participants were excluded at the exercise test/medical screening because their resting EKG's revealed cardiac abnormalities. Another individual who passed the screening was excluded because it was determined that she was physically incapable of regular exercise. Of the 267 participants who passed inclusion criteria, baseline data could not be obtained from 13 of them, four passed the stress test and provided baseline data but could not be contacted afterward regarding their group randomization and four others submitted baseline questionnaires with missing data on at least one key variable, leaving a sample of 246 for analysis in the present study.

**Figure 1:**
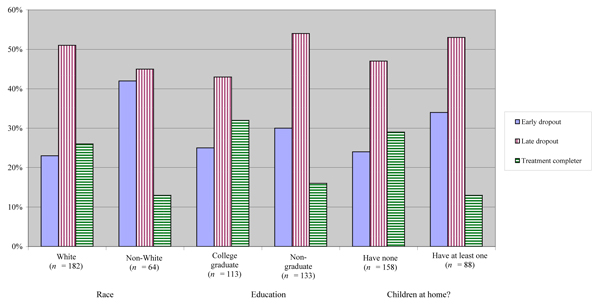
**Percentage of participants in each demographic classification who dropped out and completed treatment**.

See Figure 1 for the number of participants in each demographic classification. The 64 Non-White participants included Blacks (n = 42), Hispanics (n = 10), Asians/Pacific Islanders (n = 2) and "other" (n = 10). The 133 participants who did not graduate from college included those who had some college or technical school (n = 106), high school graduates (n = 25) and those who did not graduate from high school (n = 2). The majority of participants (55%) were single and never married, while 24% were separated/divorced/widowed and 21% were married. At the time of the trial, 78% of participants were employed full or part-time. Participants had smoked for an average of almost 21 years (M = 20.71, SD = 10.07) and their average nicotine dependence score was 4.89 (SD = 2.34), considered a moderate level of dependence based on population norms [24].

### Attrition and Demographics

Twenty-three percent of the sample completed treatment, 28% dropped out early and 49% were late dropouts. There were no significant differences in likelihood of attrition based on treatment group assignment.

See Figure 1 for the percentage of participants in each demographic classification who dropped out and completed treatment. Non-whites, those who had not graduated from college and those with at least one child living at home were more likely to have dropped out of the trial.

### Prediction of Attrition

Three pairs of predictor variables were found to be highly correlated, all at *p *< .001: age with number of years as a smoker (*r *= 0.92), depression with guilt (*r *= 0.59) and mean cigarettes smoked per day (CPD) with nicotine dependence (*r *= 0.64). A preliminary multinomial logistic regression including all variables indicated that age, guilt and CPD were stronger predictors of participation level, thus the other three variables were excluded from the main analysis.

Univariate descriptives for all continuous predictor variables included in the main regression by participation level are included in Table 1. The variables entered into this regression were found to be a good fit for the data, *X*^2 ^(24, *N *= 246) = 63.67, *p *< .001. Only one variable, having children living at home, was a significant predictor of both early and late dropout. Having at least one child living at home placed participants at higher risk of attrition. Among the predictor variables, non-white race, guilt and weight concerns were predictive of early, but not late dropout, while increasing age was a protective factor decreasing the likelihood of early dropout. Not having graduated from college was a significant predictor of late, but not early dropout (Table 2). None of the smoking-related variables included in the main analysis significantly predicted attrition.

**Table 1:** Means and Standard Deviations for Continuous Predictor Variables by Participation Level

				*Participation level*	
		Early dropout (*n *= 68)	Late dropout (*n *= 121)	Treatment completer (*n *= 57)	Total (*N *= 246)
Demographic variables	Age	36.01 (10.24)	37.80 (9.52)	39.91 (9.92)	37.80 (9.88)
	Body Mass Index (BMI)	27.15 (4.33)	26.08 (5.62)	26.10 (5.07)	26.38 (5.17)

Smoking-related variables	Cigarettes per day (CPD)	17.99 (8.34)	18.39 (8.13)	18.12 (8.32)	18.21 (8.20)
	Longest prior quit attempt (in days)	215 (413)	343 (683)	612 (982)	370 (718)
	Log transformed version of longest prior quit attempt	1.65 (0.90)	1.89 (0.86)	2.05 (1.07)	1.86 (0.93)

Psychological variables	Confidence in quit attempt (0–4 scale)	2.34 (1.05)	2.46 (0.89)	2.58 (0.93)	2.46 (0.94)
	Guilt (0–15 scale)	2.76 (2.88)	2.27 (2.37)	1.72 (1.93)	2.28 (2.45)
	Weight concern (1–24 range)	13.25 (5.82)	12.01 (5.77)	10.82 (5.73)	12.08 (5.81)

Standard deviations in parentheses

**Table 2:** Summary of Multinomial Logistic Regression Analysis Predicting Attrition (N = 246)

	Early Dropout			Late Dropout		
*Group Assignment*	*_*	*SE*	*OR (95% CI)*	*_*	*SE*	*OR (95% CI)*
Stan. Care^a^	-0.47	0.70	0.63 (0.16–2.48)	0.43	0.52	1.54 (0.56–4.29)
Wellness^a^	0.51	0.44	1.66 (0.71–3.90)	-0.32	0.39	0.73 (0.34–1.55)
*Demographics*						
Age	-0.06*	0.02	0.94 (0.90–0.99)	-0.04	0.02	0.96 (0.93–1.00)
BMI	0.00	0.04	1.00 (0.92–1.08)	-0.02	0.04	0.98 (0.92–1.06)
Children at home?^B^	1.22*	0.49	3.39 (1.31–8.78)	1.07*	0.43	2.90 (1.26–6.70)
Education ^c^	0.79	0.44	2.20 (0.94–5.19)	0.88*	0.38	2.41 (1.16–5.02)
Race^d^	1.41**	0.54	4.10 (1.41–11.87)	0.66	0.50	1.93 (0.73–5.11)
*Smoking Variables*						
Cigarettes per day	0.04	0.03	1.04 (0.99–1.10)	0.02	0.03	1.02 (0.97–1.07)
Longest quit attempt	-0.33	0.23	0.72 (0.46–1.12)	-0.13	0.20	0.88 (0.60–1.30)
*Psychological Variables*						
Confidence in quit attempt	-0.23	0.22	0.79 (0.51–1.23)	-0.19	0.20	0.83 (0.56 – 1.22)
Guilt	0.23*	0.09	1.25 (1.04–1.51)	0.16	0.09	1.17 (0.99 – 1.39)
Weight concerns	0.07*	0.04	1.08 (1.00–1.16)	0.03	0.03	1.03 (0.97 – 1.10)

a Reference group: assignment to exercise group

b Reference group: have no children living at home

c Reference group: graduated from college

d Reference group: White race

* *p *< .05 ** *p *< .01 *** *p *< .001

Results of correlation coefficients were examined in order to clarify relationships among continuous predictor variables. Participant age was positively correlated with cigarettes smoked per day (*r *= 0.36, *p *< .001) and duration of longest prior quit attempt (*r *= 0.20, *p *= .001). Guilt was positively correlated with weight concerns (*r *= 0.18, *p *= .005) and negatively correlated with confidence in the quit attempt (*r *= -0.15, *p *= .022). No other continuous predictor variables entered into the main regression were found to be significantly correlated.

Given that age and duration of longest prior quit attempt were significantly and positively correlated and that only age was found to be a significant predictor in the main analysis, a decision was made on a post-hoc basis to run a follow-up regression with age eliminated. If longest prior quit attempt was found to be a significant predictor of attrition in the absence of age, this would suggest that age acted as a proxy for this variable in the main analysis. With age omitted from the regression, length of longest prior quit attempt significantly predicted early (_ = -0.46, *SE *= 0.22, *OR *= 0.63, *95% CI*: 0.41–0.97) but not late dropout, suggesting that, at least to a degree, age acted as a proxy for this variable in the main analysis.

A series of *t*-tests were conducted in order to determine whether participants who differed in terms of race, education and having/not having children also differed with respect to any continuous variables that significantly predicted attrition in the main regression. Only one significant difference was found. Participants with children living at home (*M *= 1.68, *SD *= 2.20) had significantly lower guilt scores than those without children at home (*M *= 2.61, *SD *= 2.52), *t *(244) = 2.89, *p *= .004.

## Discussion

Attrition in the present clinical trial was predicted to a considerable extent by demographic variables. Having at least one child currently living at home was a particularly strong predictor of attrition, while Non-White race predicted early dropout and not having graduated from college predicted late dropout. That having at least one child living at home predicted attrition above and beyond educational attainment and race suggests that this may have been due to the added responsibilities faced by women with children [20]. The findings for Non-White race are in accordance with the observations of Nevid et al [16]and the added risk faced by those with lower educational attainment replicates findings reported by Borrelli et al. [2] and Curtin et al. [3]. Future research, especially involving open-ended methods, could help to clarify why women with one or more of these three risk factors are more inclined to drop out before completing participation. At the very least, researchers should be made aware that participants conforming to one or more of these demographic classifications are at a much higher risk of attrition and should be sensitive to the probability that these women face additional challenges in their efforts to remain in research studies.

Increased age was a protective factor in that the older an individual was, the less likely they were to drop out before making a quit attempt. This finding is in accordance with Fortmann and Killen's [8] observation that enrolled participants tended to be older than qualified non-participants. It is important to note that age was a significant predictor of early dropout even with length of longest prior quit attempt included in the regression, meaning that the protective quality of age was due to more than just the tendency for older participants to have had longer prior quit attempts. A replication of the effect of age on attrition and an explanation of this finding would be valuable avenues for future research.

Feelings of guilt predicted an increased likelihood of attrition. A speculative link can be made between these findings and the abstinence violation effect [23]. Those who report generally higher levels of guilt at baseline may have particular difficulty dealing with actual or anticipated "slips" during cessation treatment and choose to drop out as a result. Further research is needed to better understand relationships among guilt, the abstinence violation effect and likelihood of attrition.

That weight concerns predicted early dropout is in accordance with findings that many women who smoke, in part, do so in the attempt to maintain their weight [21,22]. Concerns about actual or anticipated weight gain apparently contributed to a decision to drop out for some women in the present trial. This finding diverges from that of Borrelli and colleagues [2], who found no effect of weight concerns on attrition.

The attrition rate for the present trial was higher than rates typically reported in the literature. The high rate of attrition could have been due to a number of factors. Our treatment protocol required attendance at a total of 24 sessions at our site in Downtown Boston for those randomized to the exercise or equal contact control conditions, in addition to at-home exercise for the exercise group and directions to use a dozen or more pieces of nicotine gum per day. The commitment required was likely seen as daunting for all but those participants who were highly motivated to quit. Those for whom the treatment was not efficacious tended to be reticent to remain in the study and attend sessions regularly. High attrition rates are not unheard of in this literature, though. In a recent 13-week trial for women featuring gum plus cognitive-behavioral group counseling, approximately one-third of participants were reported to have dropped out by the fifth week of the trial, when smoking cessation was to have been completed (Ginsberg et al., 1997). Attrition rates of over 50% are also not unusual in clinical trials testing treatments for alcohol and opiate dependence [29,30]. It has been argued that a high percentage of the smoking population is highly addicted and has been smoking for many years [29,30]. Irvin and colleagues [5,6] proposed that cessation will be increasingly difficult for this population of smokers. While the sample in the present trial can be classified as moderately nicotine dependent on average, they smoked almost a pack of cigarettes per day and had smoked for an average of over twenty years. Given the long smoking careers and heavy use of cigarettes among those in the present sample, the high rate of attrition in this trial was not entirely surprising.

Attrition is a serious problem for both researchers and participants in clinical trials. As a result, researchers are beginning to report analyses concerning attrition, but further work is needed if this problem is to be properly addressed, especially since research findings to this point have been equivocal. While all researchers should consider conducting and reporting attrition analyses, results from large, representative community samples would be particularly useful, given that the majority of published attrition findings have come from trials involving specialized samples [e.g., [2,3,16]]. Smokers dropping out of clinical trials are likely those who are most in need of assistance in quitting. Attrition therefore decreases the likelihood of successful quitting and increases the probability that smokers will eventually face morbidity and mortality. For these reasons, learning more about participants who drop out can only help to enhance cessation programs and as a result, benefit smokers, researchers and public health as a whole.

## Competing interests

The authors declare that they have no competing interests.
